# Total retinal detachments due to retinoblastoma: Outcomes following intra-arterial chemotherapy/ophthalmic artery chemosurgery

**DOI:** 10.1371/journal.pone.0195395

**Published:** 2018-04-26

**Authors:** Megan A. Rowlands, Ijah Mondesire-Crump, Ariana Levin, Audrey Mauguen, Jasmine H. Francis, Ira J. Dunkel, Scott E. Brodie, Y. Pierre Gobin, David H. Abramson

**Affiliations:** 1 Department of Medicine, Memorial Sloan Kettering Cancer Center, New York, New York, United States of America; 2 Department of Surgery, Memorial Sloan Kettering Cancer Center, New York, New York, United States of America; 3 Department of Epidemiology and Biostatistics, Memorial Sloan Kettering Cancer Center, NewYork, New York, United States of America; 4 Department of Ophthalmology, Weill Cornell Medical College, New York Presbyterian Hospital, New York, New York, United States of America; 5 Department of Pediatrics, Memorial Sloan Kettering Cancer Center, New York, New York, United States of America; 6 Department of Pediatrics, Weill Cornell Medical College, New York Presbyterian Hospital, New York, New York, United States of America; 7 Department of Ophthalmology, Mt. Sinai School of Medicine, New York, New York, United States of America; 8 Interventional Neuroradiology, Departments of Radiology, Neurosurgery and Neurology, Weill Cornell Medical College, New York Presbyterian Hospital, New York, New York, United States of America; Massachusetts Eye & Ear Infirmary, Harvard Medical School, UNITED STATES

## Abstract

**Purpose:**

To report on the rate and timing of retinal reattachment and outcomes for retinoblastoma children who have total retinal detachments at presentation to our center and were treated with intra-arterial chemotherapy (ophthalmic artery chemosurgery, OAC).

**Patients and methods:**

Single-center retrospective review of retinoblastoma patients who presented with total retinal detachments and were subsequently treated with OAC at MSKCC between May 2006 and July 2016. Endpoints were retinal detachment resolution, visual function, ERG amplitude, ocular survival, and patient survival from metastases.

**Results:**

87 eyes of 84 retinoblastoma patients were included. Using a survival multistate model, by 36 months of follow-up, there was a 54% cumulative probability of complete retinal reattachment and a 76% probability of partial reattachment. 24% of eyes that completely reattached received only OAC without any prior or adjuvant treatments. Eyes that completely reattached were significantly more likely to have been diagnosed at a younger age (p<0.0001) and to have greater initial ERG values (p = 0.006). At final follow-up, 14% of eyes had gained at least 25 μV of ERG activity, and 8.0% had achieved hand motion vision or better, including one to 20/60. 13% of eyes were enucleated. No patient died from metastatic disease, and only one developed metastases.

**Conclusion:**

OAC can successfully treat previously considered “non-salvageable” retinoblastoma eyes with total retinal detachments, promote retinal reattachment in the majority of eyes, and preserve ocular and patient survival.

## Introduction

Retinal detachment in eyes of children with retinoblastoma (RB) is common, and for most of the 20^th^ century (whether disease was unilateral or bilateral), these eyes with retinal detachments were routinely treated with enucleation.[1] While the Reese-Ellsworth classification scheme did not specifically list retinal detachment as a criterion, the majority of eyes seen worldwide were Group V, and those eyes usually had extensive retinal detachments. Reese popularized the use of external beam irradiation for retinoblastoma, but even in his hands, the majority of eyes with total detachment were primarily enucleated, and of those radiated, only half of the eyes were salvaged.[2, 3] He also felt that all eyes with unilateral retinoblastoma should be enucleated.[4]

With the popularization of systemic chemotherapy for intraocular disease, the International Classification of Retinoblastoma was developed.[5] It too did not specifically list total retinal detachment as a criterion, but many of the eyes classified as “D” and most of the “E” eyes have extensive retinal detachments.

Our group was the first to point out that salvage of eyes with total retinal detachment was possible after treatment with Ophthalmic Artery Chemosurgery (OAC),[6] and other groups have since confirmed this.[7, 8] The largest of these studies involved 37 eyes.[9] No study to date has reported on reattachment rate and details with ocular survival, patient survival, final vision and ERG results, though prior series have reported on some of these parameters. In this study, we report on these endpoints in a single center, retrospective review.

## Materials and methods

We performed a retrospective review of all retinoblastoma patients treated with OAC at Memorial Sloan Kettering Cancer Center from May 2006 to July 2016. Patients were included in our study if they had a diagnosis of retinoblastoma and, at first presentation to our center, were found to have a total retinal detachment on examination and were then subsequently treated with OAC in that eye. The charts were reviewed for details of initial presentation, International Classification of Retinoblastoma Grouping (ICRB-COG) and Reese-Ellsworth classification, the laterality of disease, family history of RB, presenting and final electroretinogram (ERG) amplitudes (30Hz flicker), prior or adjuvant intraocular or systemic treatments, and ocular and patient outcomes at the most recent follow-up. RetCam fundus photos were also reviewed in assigning each patient an ICRB group and to confirm total retinal detachment for eyes with initial ERG values > 15μV. Partial reattachment was defined as any degree of subtotal reattachment that had been appreciated on examination by our attending ocular oncologist. Retinas that re-detached during the follow-up period were coded as such, without delineating between partial or total re-detachments.

Ophthalmic artery chemosurgery (OAC) was performed in accordance with our previously published techniques.[10] In short under general anesthesia and after heparinization, a catheter was advanced from the femoral artery into the internal carotid and up to the ostium of the ophthalmic artery. After confirming the position of the catheter tip via fluoroscopy, (each) chemotherapeutic agent was injected in a pulsatile fashion at a rate of 1cc/min, over 5–10 minutes. For patients undergoing bilateral sequential OAC, the catheter was retracted and advanced to the ostium of the contralateral ophthalmic artery, position re-confirmed, and drug injected. Cerebral angiography was performed at the end of the procedure to confirm that no cerebral vascular flow anomalies were present. Patients receiving bilateral treatments or triple therapy with melphalan, carboplatin, and topotecan usually received intravenous steroids during the treatment and completed an oral steroid taper after.

Treatment doses were generally started as follows: melphalan 0.4mg/kg (3 to 7.5mg), carboplatin 30-50mg, and topotecan 0.4-2mg; these doses were adjusted at the discretion of the treating ocular oncologist and/or interventional radiologist based on extent of disease and response to treatment in addition to anatomic factors. Patients were examined under anesthesia at post-procedure week three or four, and treatments were performed at three or four week intervals as needed. Follow-up data was collected through May 2017.

We defined adjuvant treatments to be any cryotherapy, periocular chemotherapy, intravitreal chemotherapy, external beam radiation, plaque radiotherapy, or laser that occurred within 3 months of OAC. Treatments received prior to the OAC treatments at MSKCC were also included in our analyses and were limited to systemic chemotherapy and outside OAC. After presenting to MSKCC, some children were unable to receive OAC immediately, either due to low body weights or other extenuating circumstances; these children were treated with “bridge chemotherapy” (single agent systemic carboplatin) until they were able to receive OAC. As such, we considered bridge chemotherapy to be an extension of OAC. Patients who attached either partially or completely during this bridge period before OAC were excluded from our analyses.

Electroretinogram (ERG) recordings were obtained during regularly scheduled examinations under anesthesia in accordance with the International Society for Clinical Electrophysiology of Vision (ISCEV) standard protocol, which had been modified to reduce time under anesthesia, as previously described by Brodie et al.[11] A hand-held ganzfeld stimulator (Espion Colorburst, Diagnosys LLC, Lowell, MA) and ERG-jet contact lens electrode were used to record ERGs. Light adapted 3.0 single flash and 30 Hz flicker responses were obtained individually and subsequently averaged in groups of 10. These averaged waveforms were then used in our analyses.

Cumulative incidences were estimated in a competing risk setting. Time to complete reattachment was defined as the time elapsed from the date of diagnosis to the date of complete reattachment; enucleations were censored at their time of occurrence, and patients with partial reattachment or without events, i.e. reattachments or enucleation, were censored at the time of last follow-up. Similarly, time to partial reattachment was defined as the time elapsed from the date of diagnosis to the date of partial reattachment; complete reattachment and enucleation were censored at their time of occurrence, and patients without events were censored at the time of last follow-up. Risk factors for the chance of complete reattachment were studied through a survival multistate model with the following states: (1) total retinal detachment (initial state), (2) complete reattachment and (3) enucleation (absorbing state).[12] Considering the low numbers of events, the Partial Reattachment state was not included in the model. Ocular survival was defined as the time elapsed between the date of diagnosis and the date of enucleation. Patients whose eyes were not enucleated were censored at the date of last follow-up. Ocular survival was estimated using Kaplan Meier estimate. Risk factors for enucleation were studied through Cox models. Time to re-detachment was defined as the time between complete re-attachment to the date of subsequent re-detachment; enucleation was considered as a competing risk, and risk factors were studied using a multistate model. Similarly, time to phthisis was defined as the time elapsed between diagnosis and the first documentation of phthisical changes; enucleation was considered as competing event, and risk factors were studied using a multistate model.

The Memorial Sloan Kettering Cancer Center Institutional Review Board approved this study. An IRB waiver was obtained for retrospective research, which waived the requirement for written parental consent to perform this retrospective chart review; the patient information was also deidentified. This study was performed in accordance with the Declaration of Helsinki and the Health Insurance Portability and Accountability Act (HIPAA). Written consent was obtained from the patients’ parents for all procedures described.

## Results

### Demographics

**Fig 1:**
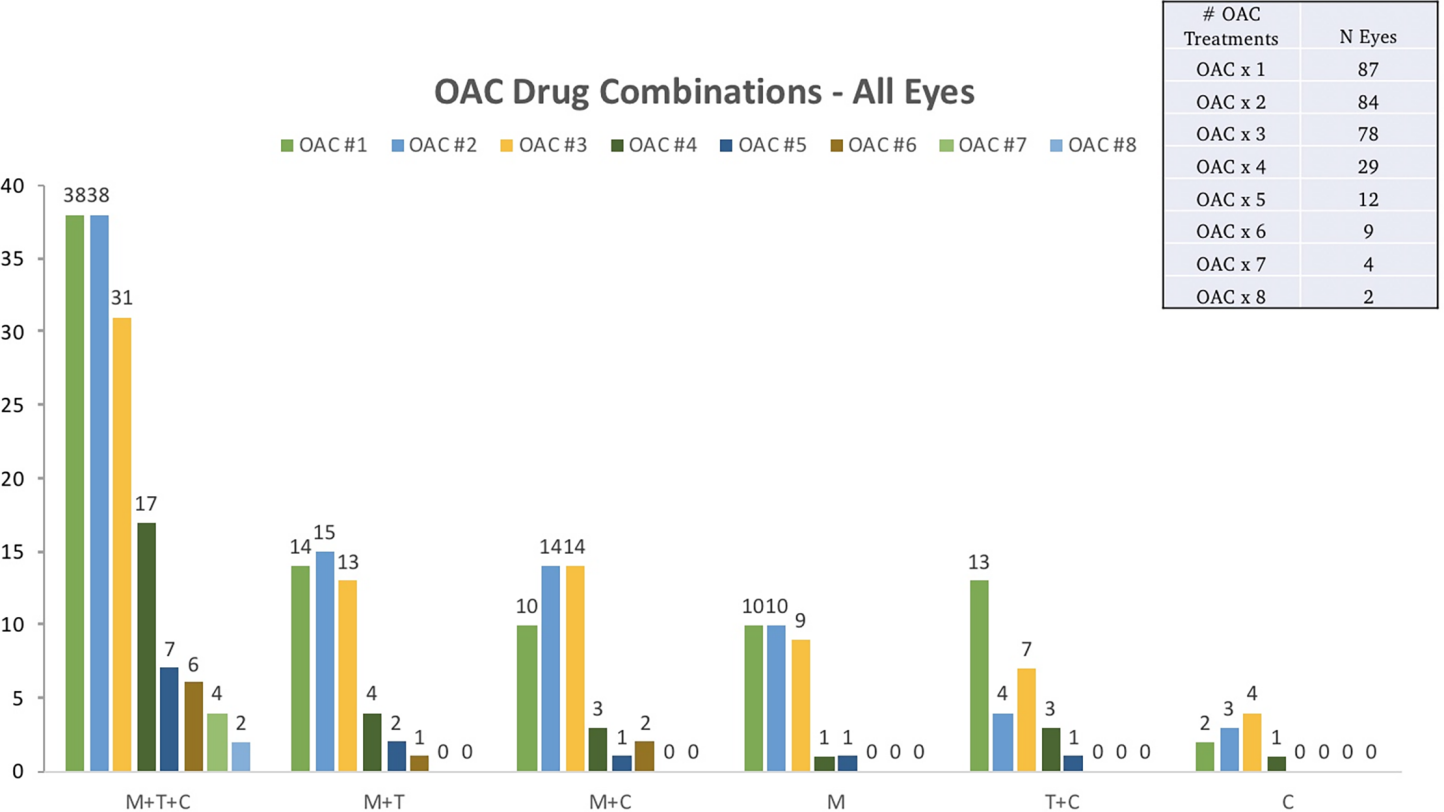
Frequency of drug treatment combinations per eye by OAC treatment. A combination of Melphalan, Topotecan, and Carboplatin was most commonly used across all OAC treatments. M: Melphalan, T: Topotecan, C: Carboplatin.

The demographics and clinical characteristics of our patients are summarized in Table 1. Eyes received a mean of 3.5 OAC treatments (median 3, range 1–8) in various combinations depending on clinical stage, whether they were naive or failed cases, whether the fellow eye was being simultaneously treated, and based on the actual vascular anatomy. The frequencies of the various combinations of OAC are shown in Fig 1. The mean time from diagnosis to the first administration of OAC was 1.6 months (range, 1 day to 12.9 months). Patients were followed for an average of 3.0 years (range 0.3–10.5 years, median 2.3 years).

**Table 1:** Clinical characteristics of study subjects.

	All 87 Total Retinal Detachment Eyes, N (%)
**Gender** (84 patients)	
** Males**	32 (38%)
** Females**	52 (62%)
**Mean age** (months)	
**At diagnosis (range)**	15.6 (0.4–60.5)
** At initial visit (range)**	16.8 (0.4–60.5)
**Bilateral retinoblastoma**	39 (45%)
**Bilateral total retinal detachments**	6 eyes (7%)
**Reese-Ellsworth Classification**	
** IIIa**	2 (2%)
** Va**	35 (40%)
** Vb**	48 (55%)
** NR**	2 (2%)
**International Classification**	
** D**	46 (53%)
** E**	40 (46%)
** NR**	1 (1%)
**Prior Treatment**	
** None**	39 (45%)
** Systemic Chemotherapy**	36 (41%)
** Intra-arterial Chemotherapy**	9 (10%)
** Bridge Chemotherapy**	3 (3%)

NR = not recorded.

Adjuvant treatments within 3 months of OAC included cryotherapy (n = 19, 22% of eyes), periocular chemotherapy (n = 7, 8%), intravitreal chemotherapy (n = 17, 20%), external beam radiation (n = 1, 1%), plaque radiotherapy (n = 4, 5%), and adjuvant laser (n = 36, 41%). Two eyes (2%) underwent retinal detachment surgery, which occurred at 3 months and at 1 year after their final OAC treatment.

### Outcomes

#### Retinal detachment resolution

Of the 87 eyes with total retinal detachments at presentation, 45 (52%) completely reattached, 15 (17%) partially reattached, and 27 (31%) remained totally detached during the follow-up period. On average, complete reattachment occurred after 2.7 OAC treatments (median 3.0, range 1–5). Of those eyes that completely reattached, 6 (13%) reattached after one OAC, 9 (20%) reattached after two OAC treatments, and 24 (53%) reattached after three; the remaining 6 (13%) required more than 3 OAC treatments. OAC drug combinations and average treatment doses for eyes that completely reattached after either one treatment, two treatments, or three treatments are listed in S1 Table. S2 Table lists the average treatment doses and combinations for all eyes, the eyes that resolved after one treatment, and those eyes that showed no resolution.

**Fig 2:**
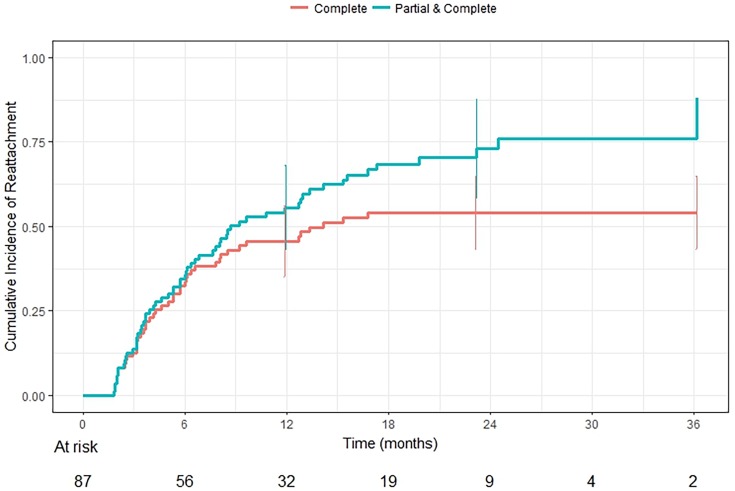
Cumulative incidence of retinal reattachment by time (months).

All retinal detachments that showed any degree of reattachment, i.e. partial or complete reattachment, did so by 36 months, and all complete reattachments occurred by 17 months. By 36 months of follow-up, there was a 54% (95% confidence interval (CI), 43 to 65%) cumulative probability of complete reattachment and a 76% (95% CI, 61 to 91%) probability of either complete or partial reattachment (Fig 2). Seventeen (37%) of those that completely reattached did so with OAC alone without any adjuvant therapy, and 11 (24%) did so without any prior or adjuvant treatment. Two eyes that completely reattached had received bridge chemotherapy. Two eyes completely reattached with OAC initially, then subsequently re-detached, and eventually underwent surgical repair for persistent detachments.

The probability of complete reattachment was higher for eyes of patients diagnosed at a younger age (p = 0.0001) and eyes with greater initial ERG values (p = 0.006). Prior systemic chemotherapy could reduce the chance of complete reattachment, but the result was not significant overall once adjusted for the other factors (Table 2).

**Table 2:** Results of the multistate survival model.

Factor	Complete Resolution (n = 45)	Partial Resolution (n = 15)	No Resolution (n = 27)	Multistate model Factors for complete resolution			
				Univariable		Multivariable	
				HR	p	HR	p
**Age at diagnosis (mo)**	11.9 +- 6.8	19.6 +- 6.8	19.6 +- 10.8	0.43 [0.90–0.97]	0.0001	0.92 [0.89–0.97]	<0.0001
**Initial ERG**	4.5 +- 7.2	2.0 +- 3.9	0.8 +- 2.7	1.06 [1.02–1.10]	0.015	1.04 [0.99–1.08]	0.006
**Previous treatment**							
***None***	27 (60%)	8 (53%)	4 (15%)	Ref	0.02	Ref	0.11
***Systemic Chemotherapy***	13 (29%)	6 (40%)	17 (63%)	0.40 [0.22–0.78]		0.40 [0.20–0.83]	
***OAC***	3 (7%)	1 (7%)	5 (19%)	0.34 [0.10–1.14]		0.52 [0.15–1.81]	
***Bridge Chemotherapy***	2 (4%)	0 (0%)	1 (4%)	0.96 [0.23–4.05]		0.57 [0.13–2.63]	
**Adjuvant Treatments**							
** Primary/Concurrent Cryotherapy**	12 (27%)	4 (27%)	3 (11%)				
** Adjuvant Periocular Chemotherapy**	0 (0%)	3 (20%)	4 (15%)				
** Intravitreal Chemotherapy**	3 (7%)	6 (40%)	8 (30%)				
** External Beam Radiation**	1 (2%)	0 (0%)	0 (0%)				
** Plaque Radiotherapy**	2 (4%)	1 (7%)	1 (4%)				
** Laser**	24 (53%)	5 (33%)	7 (26%)				

**HR**: hazard ratio; **p**: p-value; **ERG**: electroretinogram; **OAC**: ophthalmic artery chemosurgery.

Of the 45 eyes with retinas that completely reattached, 12 (27%) later re-detached. When comparing eyes that re-detached to those eyes which did not, prior therapy was more prevalent (none: 42% vs 67%, systemic chemotherapy: 50% vs 21%, OAC: 8% vs 6%, and bridge chemotherapy: 0% vs 6%, p = 0.03), while age at diagnosis (mean = 10.8 vs 12.3, p = 0.74) and initial ERG (mean = 5.2 vs 4.2, p = 0.96) were not associated with risk of re-detachment. Other factors were distributed as follows: adjuvant intravitreal chemotherapy (17% vs 3%), plaque radiotherapy (17% vs 0%), number of IA treatments (4.3 vs 3.3), adjuvant cryotherapy (42% vs 21%), EBR (8% vs 0%), or laser (67% vs 48%).

#### Visual acuity & ERG data

At initial presentation, visual acuities in the TRD eyes were no fix or follow (n = 31, 36%), no fix and follow (n = 15, 17%), fix and follow (n = 4, 4%), fix no follow (n = 1, 1%), questionably fix and follow (n = 12, 14%), questionably NLP (n = 1, 1%), or vision was not assessed (n = 23, 26%). At the final visit, 8% of eyes had HM vision or better, including one to 20/60:: 20/60 (n = 1, 1%), 20/80 (n = 1, 1%), 20/150 (n = 1, 1%), CF (n = 2, 2%), and HM (n = 2, 2%).

**Fig 3:**
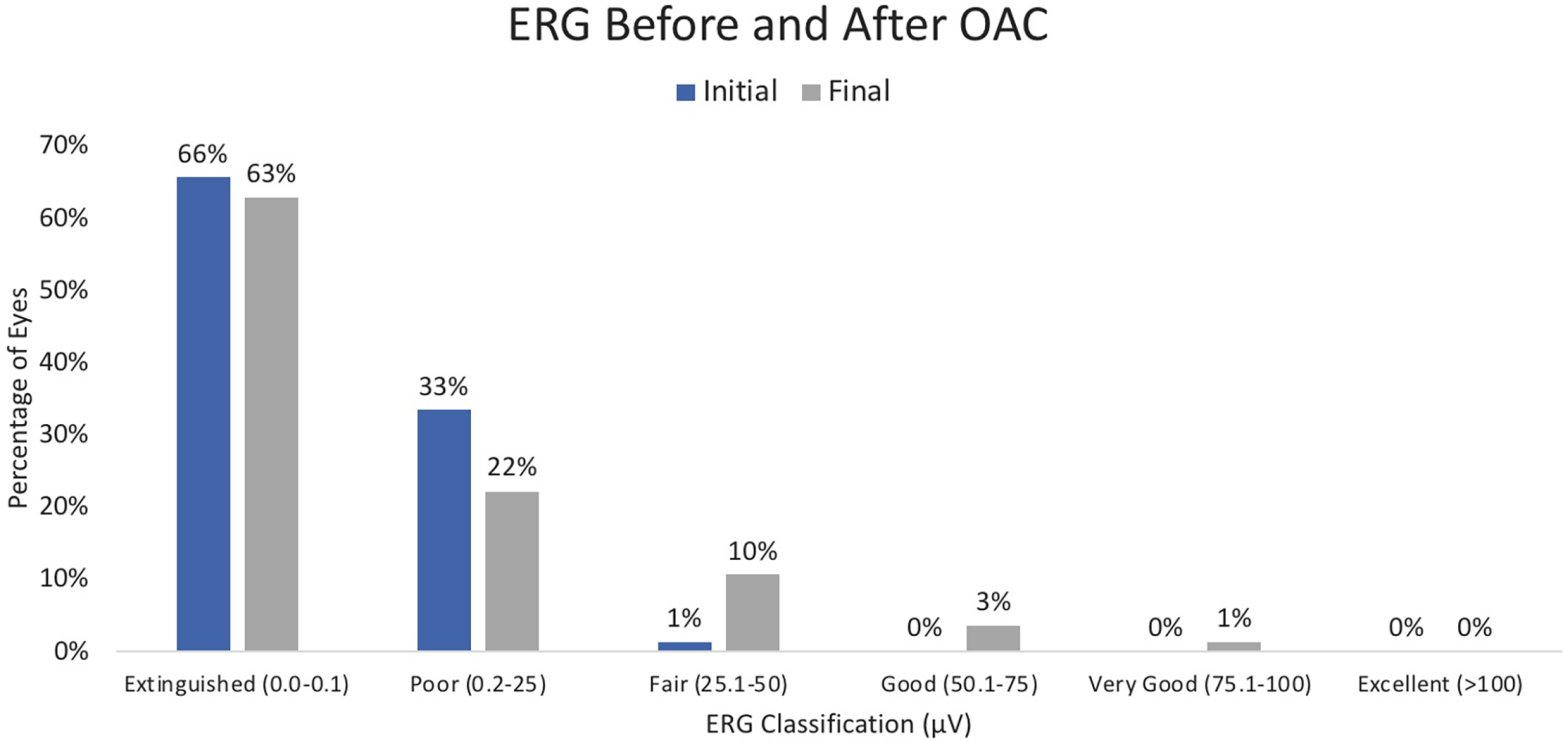
Electroretinogram (ERG) improvement after OAC.

ERG recordings were measured at initial presentation, at 3 months, 1 year, and at last follow-up (Fig 3). At initial presentation, the mean ERG value was 2.9μV (range 0–35.9, median 0). Of the 57 eyes with extinguished ERGs at initial presentation, 13 ultimately demonstrated some measurable ERG activity at the final visit (ranging from 0.95–68.3, median 15.4 μV). Of the 54 eyes with extinguished ERGs at final visit, they either had extinguished (0.0–0.1 μV) (43/54, 80%) or poor (0.20–25 μV) (11/54, 20%) ERGs on initial presentation. At the final visit, a smaller percentage of eyes were classified as poor (22% vs. 33%), and a greater percentage had developed fair (25.1–50.0 μV) (10% vs. 1%), good (50.1–75.0 μV) (3% vs. 0%), or very good (75.1–100 μV) (1% vs. 0%) ERG values. Overall, 12 (14%) eyes demonstrated a gain of at least 25 μV, while none of the eyes experienced a 25 μV loss of ERG function.

#### Ocular and patient survival

**Fig 4:**
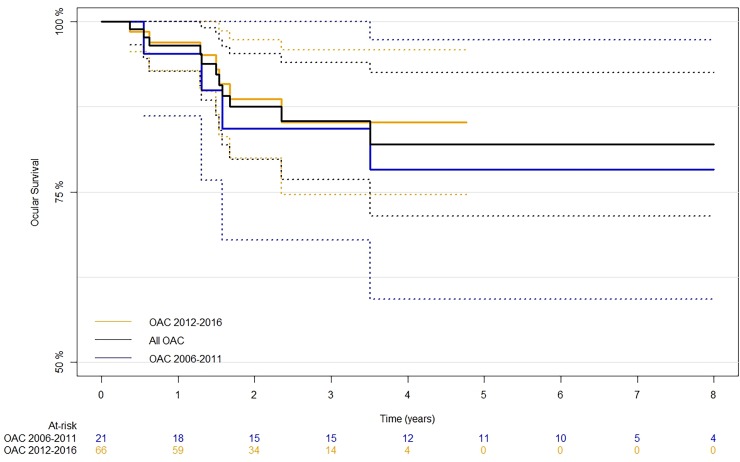
Kaplan-Meier curve of the ocular survival in OAC-treated total retinal detachment eyes, among all 87 eyes, 21 eyes treated within the first 5 years (2006–2011), and 66 eyes treated within the most recent 5 years (2012–2016). The dotted lines represent the 95% confidence interval for the survival curves.

During the follow-up, 11 (13%) of the 87 eyes were enucleated due to progression or recurrence of disease (9), pain (1), or phthisis (1). Overall, our 4-year Kaplan Meier estimate of ocular survival for the TRD eyes was 82% (95% CI, 1 to 93%, see Fig 4). Eyes that first received treatment within the most recent 5 years (2012–2016) had an 85% (95% CI, 75 to 96%) likelihood of eye retention at 4 years compared to 78% (95% CI, 59 to 97%) of eyes that had been treated during the first 5 years of OAC (2006–2011). This difference was not significant (log-rank test, p = 0.60).

There were no significant differences between enucleated and retained eyes in terms of age at diagnosis, sex, initial ERG values, or prior treatments. Fourteen (16%) of the 87 eyes became phthisical during the follow-up period. The risk of phthisis was impacted by the previous treatment (p = 0.001), with a higher risk associated with previous systemic chemotherapy (19% developed phthisis after chemotherapy vs. 10% after no treatment, HR = 4.0 [95% CI, 1.1 to 13.9]).

Overall, 24 eyes (28%) developed locally recurrent disease. None of our patients died of metastatic disease; thus, our overall metastatic patient survival rate was 100%. Only one of our patients developed metastatic retinoblastoma (to the bone marrow). Three patients developed secondary cancers (2 pineoblastomas, 1 neuroblastoma).

## Discussion

Ophthalmic artery chemosurgery is being more widely utilized as a primary treatment for retinoblastoma.[13] Total retinal detachment has long been considered an indication for enucleation, as it was thought that these eyes were non-salvageable. Our findings demonstrate that not only can the majority of these eyes be saved, but also that over half of these retinas completely reattach at least temporarily, many demonstrate measurable ERG activity, and some even acquire functional vision.

In our study, we found a 54% cumulative probability of complete reattachment among eyes with total retinal detachments after OAC and a 76% probability of eyes showing some degree of reattachment by 36 months of follow-up. Notably, 16% of eyes completely reattached after only one OAC treatment, and 86% of all eyes that completely reattached did so by three treatments. Furthermore, 24% of eyes that completely reattached did so with OAC alone, without any prior or adjuvant therapy.

This success of retinal reattachment with OAC has been reported elsewhere in the literature. In our previous work focusing on extensive (> 50%) bullous, serous retinal detachments in 37 RB eyes, we found that 76% of eyes reattached, with 58% of reattachments occurring within 3 months and three OAC infusions.[9] Shields et al. also observed resolution of total retinal detachments with OAC. Among 7 eyes with total retinal detachments, three (43%) of these eyes completely resolved, two (29%) partially resolved, and two (29%) showed minimal improvement. Of the eyes with partial detachments, 100% resolved during treatment.[8] In our previous study of advanced RB eyes with baseline ERGs that were either extinguished or “poor” (< 25 μV), OAC alone was successful in reattaching 59% of the eyes with baseline retinal detachments within 3 months of initiation.[14]

Of note, we did not compare the rates of reattachment after OAC to reattachment after systemic chemotherapy or radiation alone, in large part because we do not have any eyes that have been treated solely with these modalities. The vast majority (> 90%) of advanced eyes at our center are not enucleated but rather are treated with OAC.[15] Interestingly, prior chemotherapy appeared to reduce reattachment rates in the univariate analysis, but this was not significant when adjusted for other factors. It would be interesting to further investigate this by looking at rates of reattachment among all total retinal detachment eyes that had received chemotherapy. In our case, however, the majority of patients who received systemic chemotherapy did so at outside institutions, and we could not always be certain of the original status of the retina. Thus, we opted to only include patients who presented to our center with a total retinal detachment that was confirmed on our examination.

In the present study, patients whose eyes completely reattached were significantly younger at initial diagnosis (average 11.9 months) and had higher initial ERG values. The fact that many of our TRD eyes had initial ERG values that were greater than zero suggests that these eyes were more shallowly detached in areas, which may have facilitated their reattachment later. There was no significant difference in reattachment between eyes that had received previous treatment compared to those that had not, including prior OAC outside of MSKCC. Some of the adjuvant treatments often used for retinoblastoma, e.g. radiation, cryotherapy, laser, have been shown to predispose to retinal breaks.[16] We did not perform statistical analyses to assess for significance of adjuvant treatments because the timing of these interventions varied between patients. However, a larger percentage of eyes that completely reattached had received adjuvant cryotherapy (27% vs. 11%) and laser (53% vs. 26%) compared to those that did not, which suggests that these treatments at least do not seem to hinder reattachment.

27% of our eyes that had achieved complete reattachment subsequently re-detached, many of which occurred months to years after completely reattaching. Of note, these eyes that re-detached were significantly more likely to have received treatment prior to OAC than those that remained attached, which may indicate that these eyes had more advanced or persistent disease and thus required additional therapy, or it may reflect cumulative toxicity when these additional interventions were employed.

Our study also illustrates that total retinal detachment eyes with extinguished or poor retinal function prior to OAC are capable of recovery of ERG amplitude and measurable visual acuity, but most remained (as they were at presentation) poor or extinguished at final visit. However, some of these eyes demonstrated large gains in ERG function (range 0.95–68.32μV). After OAC, 14% of our TRD eyes ultimately achieved “fair”, “good”, and “very good” ERG activity. Although improvements in ERG activity did not necessarily correspond to improvements in vision, it is encouraging that even these severely damaged retinas are capable of some degree of functional recovery.

Thus, our study suggests that even TRD eyes with extinguished ERG amplitudes at initial presentation are still suitable candidates for receiving OAC and may in fact demonstrate the greatest gains in ERG function. In our previous work involving some of our most advanced (Reese-Ellsworth Va or Vb) patients treated with OAC, we had similarly observed improvements in ERG function, which correlated with resolution of retinal detachments.[9, 17] This was also noted by Abdelhakim et al.[14]

In this study, our 4-year Kaplan Meier estimate of ocular survival was calculated as 82%, which is similar to the 79% of group D eyes that had been saved in our previous study.[18] Notably, we found that patients treated within the most recent five years had greater ocular survival rates (85%) compared to our cohort treated within the first five years (78%), although this difference was not significant. Reassuringly, attempting to treat these “hopeless” eyes has not resulted in metastatic deaths.

Our study has multiple limitations. For one, since the patients in this study were followed through May 2017, some patients only had approximately 10 months of follow-up data recorded, and the statuses of their retinas may have changed after the follow-up period ended. Secondly, our OAC drug combinations and dosages varied between patients and treatment sessions, which limits our ability to interpret or correlate treatment regimens on outcomes. Also, treatments were not limited to OAC alone, and many of our patients had received previous or adjuvant treatments in addition to OAC. Additionally, our definition of partial reattachment included any degree of reattachment even if the majority of the retina remained detached, and we did not attempt to quantify or stratify the amount of partial reattachment. Finally, since multiple statistical analyses were performed, we must be cautious in our interpretation of significance based on our p-values.

## Conclusions

In this study, we investigated the effect of OAC on total retinal detachments associated with retinoblastoma. Over half of our eyes with total retinal detachments achieved complete reattachment, and the vast majority showed some degree of partial reattachment. Furthermore, some of these eyes ultimately regained both measurable ERG function and some vision, including one whose vision improved to 20/60. Prior to the advent of OAC, these eyes were considered “non-salvageable” and would have undergone primary enucleation without question. It is reassuring that attempting to treat eyes with total retinal detachment did not compromise patient survival from metastatic disease.

## Supporting information

S1 Table OAC drug combinations and average treatment doses (in mg) used in the eyes that reattached after 1 OAC treatment (n = 6 eyes), after 2 treatments (n = 9 eyes), and after 3 treatments (n = 24 eyes). M = melphalan, C = carboplatin, T = topotecan.
(PDF)

S2 Table This table shows the first OAC treatment and average doses (in mg) for all eyes, for the eyes that reattached after one treatment, and for the eyes that had no resolution. M = melphalan, C = carboplatin, T = topotecan.
(PDF)

S1 Dataset (XLSX)
