# Supplementary material for: Total retinal detachments due to retinoblastoma: Outcomes following intra-arterial chemotherapy/ophthalmic artery chemosurgery
Source: PLoS One. 2018 Apr 26;13(4):e0195395. doi: 10.1371/journal.pone.0195395 (PMC5919618; doi:10.1371/journal.pone.0195395)
Supplement: S1 Table — M = melphalan, C = carboplatin, T = topotecan. (PDF) [file pone.0195395.s001.pdf]

	**OAC # 1**					**OAC # 2**					**OAC # 3**				
**Resolution after 1 OAC Treatment**		#	Avg. M	Avg. T	Avg. C										
	M	2	2.8 ± 0.4	-	-										
	C	0	-	-	-										
	M+T	1	2.5 ± 0.0	0.3 ± 0.0	-										
	M+C	0	-	-	-										
	M+T+C	1	3.0 ±0.0	1.0 ± 0.0	30 ± 0.0										
	T+C	2	-	0.5 ± 0.1	30 ± 0.0										
**Resolution after 2 OAC Treatments**		#	Avg. M	Avg. T	Avg. C		#	Avg. M	Avg. T	Avg. C					
	M	2	4.0 ± 1.4	-	-	M	2	4.0 ± 1.4	-	-					
	C	0	-	-	-	C	1	-	-	40 ± 0.0					
	M+T	2	3.0 ± 0.0	0.7 ± 0.5	-	M+T	1	3.0 ± 0.0	1.0 ± 0.0	-					
	M+C	0	-	-	-	M+C	0	-	-	-					
	M+T+C	4	3.6 ± 1.0	1.3 ± 0.9	40 ± 12	M+T+C	4	3.6 ± 1.0	1.3 ± 0.9	40 ± 12					
	T+C	1	-	0.5 ± 0.0	40 ± 0.0	T+C	1	-	0.3 ± 0.0	30 ± 0.0					
**Resolution after 3 OAC Treatments**		#	Avg. M	Avg. T	Avg. C		#	Avg. M	Avg. T	Avg. C		#	Avg. M	Avg. T	Avg. C
	M	2	3.5 ± 0.7	-	-	M	3	3.0 ± 1.0	-	-	M	4	3.6 ± 1.1	-	-
	C	3	-	-	45 ± 7.1	C	2	-	-	50 ± 0.0	C	2	-	-	50 ± 0.0
	M+T	4	2.6 ± 0.5	1.0 ± 0.8	-	M+T	6	3.0 ± 0.3	0.8 ± 0.7	-	M+T	5	3.0 ± 0.0	0.7 ± 0.7	-
	M+C	4	3.6 ± 0.5	-	45 ± 5.8	M+C	6	3.8 ± 0.4	-	53 ± 14	M+C	5	3.7 ± 0.5	-	48 ± 4.5
	M+T+C	7	3.1 ± 0.4	1.0 ± 0.7	35 ± 12	M+T+C	6	3.3 ± 0.5	1.2 ± 0.7	40 ± 13	M+T+C	4	3.5 ± 0.6	1.6 ± 0.8	45 ± 13
	T+C	5	-	1.0 ± 0.7	47 ± 16	T+C	1	-	0.5 ± 0.0	50 ± 0.0	T+C	4	-	1.1 ± 0.6	58 ± 17

Supplemental table 1
