# Supplementary material for: Total retinal detachments due to retinoblastoma: Outcomes following intra-arterial chemotherapy/ophthalmic artery chemosurgery
Source: PLoS One. 2018 Apr 26;13(4):e0195395. doi: 10.1371/journal.pone.0195395 (PMC5919618; doi:10.1371/journal.pone.0195395)
Supplement: S2 Table — M = melphalan, C = carboplatin, T = topotecan. (PDF) [file pone.0195395.s002.pdf]

All Eyes					Resolution after 1 OAC Treatment					No Resolution				
	#	Avg. M	Avg. T	Avg. C		#	Avg. M	Avg. T	Avg. C		#	Avg. M	Avg. T	Avg. C
M	10	3.6 ± 0.9	-	-	M	2	2.8 ± 0.4	-	-	M	0	-	-	-
C	2	-	-	45.0 ± 7.1	C	0	-	-	-	C	0	-	-	-
M+T	14	3.1 ± 0.9	0.6 ± 0.5	-	M+T	1	2.5 ± 0.0	0.3 ± 0.0	-	M+T	2	3.0 ± 0.0	0.8 ± 0.4	-
M+C	10	4.0 ± 1.4	-	41.0 ± 7.4	M+C	0	-	-	-	M+C	4	4.3 ± 2.3	-	38 ± 9.6
M+T+C	38	3.9 ± 0.9	1.3 ± 0.7	42.2 ± 14.2	M+T+C	1	3.0 ±0.0	1.0 ± 0.0	30 ± 0.0	M+T+C	17	4.3 ± 1.0	1.1 ± 0.6	42 ± 16
T+C	13	-	0.8 ± 0.5	47.3 ± 15.4	T+C	2	-	0.5 ± 0.1	30 ± 0.0	T+C	4	-	0.8 ± 0.3	58 ± 15

Supplemental table 2
